# Omentin-1 plasma levels and cholesterol metabolism in obese patients with diabetes mellitus type 1: impact of weight reduction

**DOI:** 10.1038/nutd.2015.33

**Published:** 2015-11-02

**Authors:** J Lesná, A Tichá, R Hyšpler, F Musil, V Bláha, L Sobotka, Z Zadák, A Šmahelová

**Affiliations:** 1 3rd Department of Internal Medicine, University Hospital in Hradec Králové, Hradec Králové5, Czech Republic; 2 Centre for Research and Development, University Hospital in Hradec Králové, Hradec Králové, Czech Republic; 3 Department of Clinical Biochemistry, University Hospital in Hradec Králové, Hradec Králové5, Czech Republic; 4 Medical Faculty in Hradec Králové, Charles University in Prague, Hradec Králové1, Czech Republic

## Abstract

**Background:**

Omentin-1 is an anti-inflammatory adipokine produced preferentially by visceral adipose tissue. Plasma levels of omentin-1 are decreased in obesity and other insulin-resistant states. Insulin resistance contributes to the changes of cholesterol synthesis and absorption as well. The aim of this study was to characterise omentin-1 plasma levels in obese patients with diabetes mellitus type 1 during weight reduction, and to elucidate the relationship between cholesterol metabolism and omentin-1.

**Methods:**

Plasma levels of omentin-1 were measured in obese type 1 diabetics (*n*=14, body mass index >30 kg m^−2^, age 29–62 years) by enzyme-linked immunosorbent assay (BioVendor). Gas chromatography with flame ionisation detector (Fisons Plc.,) was used to measure squalene and non-cholesterol sterols—markers of cholesterol synthesis and absorption (phase I). Measurements were repeated after 1 month (phase II; 1 week of fasting in the hospital setting and 3 weeks on a diet containing 150 g saccharides per day) and after 1 year (phase III) on a diet with 225 g saccharides per day.

**Results:**

Omentin-1 plasma levels were stable during phases I and II, but significantly increased (*P*<0.001) during phase III. Omentin-1 plasma dynamics were significantly associated with plasma levels of high-density lipoprotein (*P*=0.005) and triacylglycerols (*P*=0.01), as well as with lathosterol (*P*=0.03).

**Conclusion:**

Omentin-1 plasma levels significantly increased during the weight reduction programme. Omentin-1 plasma dynamics suggest a close relationship with cholesterol metabolism.

## Introduction

Obesity is typically related to diabetes mellitus type 2, although it has become a serious problem even among type 1 diabetics.^1^ Diabetes mellitus type 1 is a chronic disease characterised by absolute lack of insulin, resulting from autoimmune destruction of the pancreatic β-cells. This malfunction interferes not only in carbohydrate metabolism but also in that of fat and protein, and leads to a wide range of multisystem abnormalities with a common etiopathogenesis of formidable macro- and microvascular disease.^2^

In obese diabetics, redundant fat tissue is associated with insulin resistance, dyslipidemia, hypertension, endothelial dysfunction and a pro-inflammatory state. Obesity can thus markedly contribute to the development of diabetic complications. In these effects adipose tissue-secreted cytokines/adipokines are implicated.^3, 4^

Omentin-1 (intelectin-1, intestinal lactoferrin receptor, endothelial lectin HL-1, galactofuranose-binding lectin), a 313-amino acid peptide, is an anti-inflammatory adipokine preferentially expressed in stromal vascular cells of visceral adipose tissue. It is suggested that this substance makes an important contribution to the physiological difference between visceral and subcutaneous adipose tissue. It is abundant also in human vasculature, the small intestine, colon, thymus and heart.^5^ Omentin-1 is the major circulating form; it also has a homologue designated as omentin-2 (ref. 6) and their genes are localised adjacent to each other at 1q22-q23 in the region linked to diabetes mellitus type 2. Both omentin homologues in circulating form correlate with expression in visceral fat tissue.^7^ Omentin-1, as with adiponectin, can activate 5′-AMP-activated protein kinase and endothelial nitric oxide synthase.^8, 9^ Via this activation, omentin-1 is essentially involved in cellular energy homoeostasis and vascular tone regulation. Omentin-1 increases insulin signal transduction, enhances insulin-stimulated glucose transport in human adipocytes (but has no effect on basal glucose uptake) and contributes to regulation of lipid metabolism.^6^ In contrast to adiponectin, omentin-1 can inhibit activation of JNK (c-Jun N-terminal kinases), and thus it is suggested that omentin-1 is involved in stress responses, expression of heat shock proteins, T-cell differentiation and apoptosis.^6, 10^

Omentin-1 is known to modulate immune reactions of the organism, and thus may have an anti-inflammatory effect, a significant association with inflammatory markers has been reported.^11, 12^ It takes part in defence mechanisms by binding to galactofuranoses on bacteria. Omentin-1 inhibits the TNF-α mediated induction of pro-inflammatory molecules in vascular endothelial cells. Important roles have also been suggested in vasodilatation, development of endothelial dysfunction and arterial calcification.^13, 14, 15, 16^ Plasma levels of omentin-1 are decreased in insulin-resistant and pro-inflammatory states (diabetes mellitus type 1 and 2, obesity, polycystic ovary syndrome and so on).^10, 17^ Obesity and insulin-resistant states are associated with enhanced endogenous cholesterol synthesis as well, cholesterol absorption remains lower in comparison with non-insulin-resistant population.^18, 19^ In obese patients plasma omentin-1 levels increase after weight loss;^20^ however, dynamics of plasma omentin-1 and markers of cholesterol metabolism in obese type 1 diabetics have not been characterised so far. Possible insulin-sensitising and anti-inflammatory effect of omentin-1 could have a positive role in changes of cholesterol metabolism in diabetes mellitus type 1.

In the present study, we hypothesised an increase in plasma omentin-1 levels after a weight reduction programme in obese patients with diabetes type 1. Association with markers of cholesterol endogenous synthesis was hypothesised. To examine the short- and long-term effects of weight reduction, we observed patients over the course of 1 year.

## Materials and methods

### Recruitment of patients

The study group of obese patients with diabetes mellitus type 1 (*n*=14, body mass index (BMI) >30 kg m^−2^, age 29−62 years, male/female ~9/5) was recruited from the register of diabetics of University Hospital in Hradec Králové. Inclusion criteria were as follows: diabetes mellitus type 1 (diagnosed at least 1 year ago and C-peptide=below detection limit), BMI >30  kg m^−2^, age 20–70 years and supposed good compliance. Exclusion criteria were as follows: gravidity, serious comorbidity (cancer, heart or renal failure and so on) and non-compliance. Plasma levels of omentin-1 in phase I were compared with the not-intervened subgroup of obese patients with diabetes mellitus type 2 (*n*=21, BMI >kg m^−2^, age 39–69 years).

The study was approved by the local Ethics Committee in Hradec Králové, Czech Republic. All patients signed informed consent before the study. ENCePP Register of Studies: ENCEPP/SDPP/8455.

### Clinical procedure

The study programme for weight reduction commenced with a stay in hospital for 7 days. On the first day, patients were admitted in the afternoon and began fasting. Next morning, weight, height and waist circumference were measured, and BMI was calculated as body weight (kg) divided by height (m) squared. Waist circumference was measured in half distance between ribs and iliac bones. Body composition measurement was carried out using a body composition monitor (Fresenius Medical Care, Bad Homburg (vor der Höhe), Germany). Blood samples were collected, and after centrifugation the resulting plasma samples were kept frozen at −20 °C till further analysis (phase I).

During the 7 days of hospital stay the patients were fasting, using only basal doses of daily insulin. Peroral intake of patients during the 7 days of fasting was minimised. Patients used hypotonic drink POWERline (Isoline, Prague, Czech Republic) 1 l per day, B-komplex (Zentiva, Prague, Czech Republic) 1 tablet per day, Celaskon (Zentiva) 1 tablet per day and Anacid (TEVA, Prague, Czech republic) three suspensions per day. Glucose metabolism was controlled by regular checks of glycaemia (four times a day) and blood ketone bodies (two times a day). During the hospital stay, patients were educated to a diet with 150 g of saccharides per day (1200 kcal per day: 150 g saccharides, 75 g protein and 40 g fat), which they maintained for the next month even after discharge. At the outpatient visit after a month, the previously mentioned measurements were repeated (phase II). The study group then continued for a further 11 months on a diabetic diet (1650 kcal per day: 225 g saccharides, 75 g protein and 50 g fat; phase III). Frequent consultations were planned to encourage patients in the following nutritional and rational regimen recommendations. Measurements were taken in all three following phases: before the reduction programme and 1 month and 1 year after the hospital setting.

### Laboratory tests

Blood was collected in the fasted state. Total cholesterol, high-density lipoproteins, low-density lipoproteins and triacylglycerols (TAG) were measured enzymatically (Modular Analytics, Roche, Basel, Switzerland). Squalene and non-cholesterol sterols (lathosterol—marker of cholesterol synthesis; campesterol and β-sitosterol—markers of cholesterol absorption) were measured by gas chromatography with a flame ionisation detector (Fison Plc., Great Britain, UK). After centrifugation, plasma samples were kept frozen at −20 °C till further analysis. Free fatty acids were analysed with a free fatty acids-HR kit (Wako chemicals GmbH, Neuss, Germany) using a ultraviolet−visible spectrophotometer (Shimadzu Pharma Spec 1700 UV Probe, Kyoto, Japan). Plasma levels of omentin were measured in obese type 1 diabetics by enzyme-linked immunosorbent assay (ELISA, BioVendor, Heidelberg, Germany).

Data were statistically analysed by software Sigma Stat—one-way analysis of variance with repeated measures. Data are presented as median (25% and 75%).

## Results

All subjects completed the study programme. We registered no health complication (including serious hypoglycaemia) during the reduction programme.

### Clinical parameters and laboratory results

The detailed characteristics of the study group is given in Table 1. Three patients of the study group used statin (atorvastatin 20 mg per day); however, the dose was not changed during the weight reduction programme. No patient used hormonal contraceptives or other hormonal therapy.

During the weight reduction programme, the BMI and waist circumference of the obese type 1 diabetics significantly decreased (phases I vs II and I vs III). Daily doses of insulin were lowered from 50.0 (39.25; 54) IU in phase I to 42.0 (39.3; 51.0) IU in phase III (*P*<0.001); however, HbA1c (glycated haemoglobin, International Federation of Clinical Chemistry) significantly (*P*<0.05) increased from 5.8 (5.45; 6.55) % to 6.6 (6.08; 6.93) %.

A significant decrease (*P*<0.05) in total cholesterol and low-density lipoproteins cholesterol was detected in phases I vs II (Table 1). Markers of cholesterol synthesis and absorption were significantly lowered (*P*<0.05) during this period.

Mild positive changes in lipid profile and cholesterol metabolism markers were detected between phases I and III (Table 1). A significant decrease in lathosterol and the lathosterol/cholesterol ratio (markers of endogenous cholesterol synthesis) was observed (*P*<0.001). Similarly, plasma levels of campesterol as well as the campesterol/cholesterol ratio (markers of cholesterol absorption) were significantly lowered from phase I to phase III of the reduction programme. Plasma levels of free fatty acids tended to decrease.

No plasma omentin-1 differences were found between the men and women subgroups. Significantly lower levels (*P*=0.032) of omentin-1 in not-intervened control subgroup of type 2 diabetics (BMI=35.1 (32.3; 37.1); omentin=2.15 (1.32; 6.02)) in comparison with phase I in diabetes mellitus type 1 were detected (Table 2). Comparison of basic characteristic of study group (obese diabetics type 1) and control group (obese diabetics type II) is given in Table 2. Significant differences were found in compensation of diabetes mellitus and body composition (Table 2).

**Figure 1:**
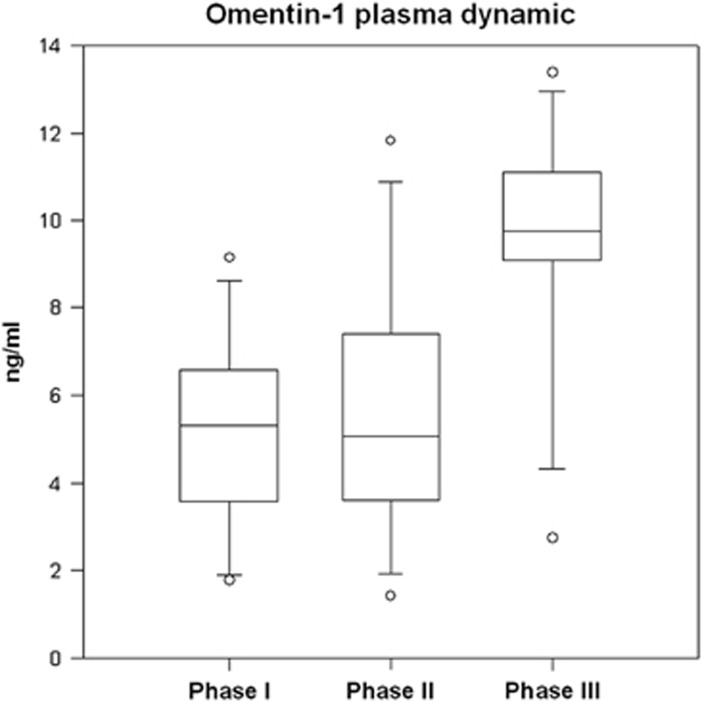
Omentin-1 plasma dynamic during the programme in obese type 1 diabetics.

Omentin-1 plasma levels in type 1 diabetics (Figure 1) were stable during phases I and II ((5.31 (3.72; 6.49)–5.05 (3.91; 7.32) ng ml^−1^), but was significantly increased in phase III (9.74 (9.11; 10.98) ng ml^−1^).

In patients with diabetes mellitus type 1 no association of omentin-1 plasma level dynamics with BMI, HbA1c, free fatty acids or body composition measurement was found.

Plasma omentin levels were significantly associated with plasma levels of high-density lipoproteins (*r*=0.44, *P*=0.005) and TAG (*r*=−0.41, *P*=0.011; Table 3). Association was also noted with the markers of cholesterol synthesis (lathosterol (*r*=−0.34, P=0.033), lathosterol/cholesterol ratio (*r*=−0.32, *P*=0.05)) and absorption (campesterol (*r*=−0.32, *P*=0.05; Table 3)).

Changes in metabolic parameters were correlated with changes of plasma levels of omentin-1 between phases I and II, II and III, and I–III. No significant associations were found in phases I and II, and II and III. In phases I–III, significant association of omentin-1 changes with the changes in glycated haemoglobin (*r*=−0.72, *P*=0.008) and TAG (*r*= −0.64, *P*=0.022) was found (Table 4).

## Discussion

Omentin-1 is an important anti-inflammatory adipokine produced preferentially in visceral adipose tissue. It has been reported that circulating levels of omentin-1 are related to insulin resistance, obesity, dyslipidemia, endothelial dysfunction, arterial hypertension and so on.^21, 22^ The objective of this study was to examine plasma omentin-1 dynamics in obese patients with diabetes mellitus type 1 during the weight reduction programme. We focused on the relationship between changes in omentin-1 levels, the lipid spectrum and markers of cholesterol metabolism.

During the weight reduction programme, BMI of the study group decreased significantly (phases I and II, and I–III). The percentage of fat and lean tissue changed non-significantly, fat tissue index decreased significantly in phases I and II. These results correspond with the dietary and regime change efforts of the study group.

The therapeutic daily insulin dose of the study group was significantly lowered. This was partially driven by patients' concerns about hypoglycaemia during the weight reduction. Although following the diet and regime recommendations in their home conditions, some patients applied rather less insulin than was needed and recommended by the study doctor. This could be related to the significant increase in HbA1c (phases I–III), the marker of long-term diabetic compensation. However, no increase in hypoglycaemia events was recorded during the weight reduction programme. Another reason for worsening of diabetic compensation could be related to increased physical activity itself. According to our clinical experience, increased physical effort in diabetic patients on insulinotherapy is quite often related to greater variability in glycaemic profile. Increased glycaemic variability can tend to worsened compensation (especially during the initial months of regime changes). Therefore, we can speculate this mechanism that could contribute to worsened HbA1c in patients who increased physical activity effort markedly (this suggestion corresponds well with case history data of some patients). On the other hand, in several patients worsened compliance was noticed at the end of the reduction programme, in comparison to their effort at the beginning of the reduction programme. This could contribute to significant increase in glycated haemoglobin as well.

Mild changes in lipid profile were detected in phases I–III of the reduction programme. Total cholesterol, low-density lipoproteins and TAG tended to decrease, whereas plasma levels of high-density lipoproteins increased significantly (*P*<0.05). These changes were expressed especially in well-compliant individuals who managed to increase mildly their daily physical activity. The dynamic in plasma levels of non-cholesterol sterols and squalene suggests long-term changes in cholesterol metabolism. The decrease in campesterol/cholesterol ratio signifies lowered cholesterol absorption, which corresponds with rational diet recommendations (phases I and II, and I–III). The significant decrease in lathosterol and the lathosterol/cholesterol ratio in phase III indicates lowered endogenous synthesis of cholesterol. These dynamics could be evidence for long-term changes in cholesterol metabolism closely related to insulin resistance. Increase in insulin sensitivity is accompanied by raised activity of 5′-AMP-activated protein kinase, which is known to inhibit endogenous cholesterol synthesis.

Omentin-1 plasma levels in the study group were generally lower than those measured in most of the other studies for obese groups with or without diabetes mellitus type 2 (refs. 20). Tan *et al.* ^17^ found that plasma omentin-1 levels were decreased in subjects with type 1 diabetes. Obese patients with diabetes mellitus type 1 suffer from primary absolute insulin deficiency, related itself to lower omentin-1 levels. Furthermore, they have increased insulin resistance because of their obesity. This lowers the effect of therapeutic insulin, which is typical for diabetes mellitus type 2. Thus, we speculate that this ‘double diabetes' of the study group could be the cause of very low omentin-1 plasma levels in our study group. In contrast to this fact, we found significantly lower plasma levels of control non-intervened group of patients with diabetes mellitus type 2; however, we expected lower levels during phase I in subgroup of type 1 diabetics. Differences in the age of study subgroups (higher age in patients with diabetes mellitus type 2) and body composition (increased percentage of fat tissue in subgroup of patients with diabetes mellitus type 2) could possibly contribute to these results, as we know both are related to increased insulin resistance. However, these comparison needs to be proved in larger study group of diabetic patients.

Many adipokines have been found to exhibit a sexual dimorphism. Although omentin-1 dimorphism has been reported in some studies,^20, 24^ in our study group no differences between the men and women subgroups were found. Tan *et al.*^17^ reported no significant differences in omentin-1 net protein production during investigation of steroid effects *ex vivo* (except for a negative association with 17β-estradiol).^11^ However, speculated omentin-1 sexual dimorphism is not expected to have a significant role in this study, because there is no difference in plasma omentin-1 dynamics following regime changes in the male and female subgroups.^20^

Other studies^7, 10, 23^ have suggested a correlation of omentin-1 levels with BMI, fat tissue mass (FTM) and HbA1c. In our study, plasma omentin-1 levels were stable in phases I and II, although the body weight and fat tissue mass of the study patients rapidly decreased during this period. The plasma omentin-1 level dynamic does not follow BMI or FTM in short-term changes. In phase III, a significant increase in omentin-1 levels was detected, even when the BMI, FTM and lean tissue mass of the diabetics did not change significantly compared with phase II (Table 1). We found no association of omentin-1 plasma level dynamics with BMI or fat tissue amount. Negative association between changes in plasma omentin and glycated haemoglobin was found in phases I–III.

Significant increase in plasma omentin-1 levels was detected in phase III, which could indicate a long-term regulation of omentin-1 expression. According to our data, plasma omentin-1 levels do not simply follow body weight or amount of fat or lean tissue, but they change more likely in relation with overall reorganisation of fat tissue caused by the long-term regime and diet changes. This is supported by findings in other studies, where no significant dynamic in plasma omentin-1 was reported during acute intervention (for example, glucose load),^23^ whereas a significant increase in omentin-1 plasma levels was detected after several weeks of regime changes, such as regular aerobic training in obese patients^25^ or low-calorie diet.^20^ However, other studies concede short-term changes in omentin-1 plasma levels after prolonged insulin–glucose infusion.^10^

Omentin-1 plasma levels decrease in insulin-resistant states.^26, 27^ In our study, the long-term observation showed significantly higher plasma omentin-1 levels, which could be indicative of increase in insulin sensitivity. However, Tan *et al.*^12^ reported that hyperinsulinemia significantly reduced plasma omentin-1 levels in healthy subjects, which is why we could speculate that the reverse mechanism could contribute to elevated omentin-1 levels after daily doses of insulin had been artificially lowered. In contrast, insulin daily dose was rapidly lowered already during phase II, whereas omentin-1 plasma levels remained stable.

A positive association of omentin-1 plasma levels with high-density lipoproteins and a negative association with TAG were found, which concurs with other studies^22^ (Table 3). Significant negative association between TAG changes and the changes in plasma omentin-1 was found in phases I–III. Furthermore, we are first to show a negative association of omentin-1 with the markers of endogenous cholesterol synthesis—lathosterol and the lathosterol/cholesterol ratio. However, degree of changes in these parameters between particular phases do not seem to be associated together.

Omentin-1 can activate 5′-AMP-activated protein kinase,^28, 29, 30^ which works as a powerful endogenous cholesterol synthesis inhibitor, and thus we speculate that omentin-1 could contribute to regulation of cholesterol synthesis, via this pathway. However, the exact relation to cholesterol metabolism is still unclear. There is thus a wide field for future research.

## Conclusion

In conclusion, the present study has primarily characterised the dynamic of plasma omentin-1 during the weight reduction in patients with diabetes mellitus type 1. Furthermore, the presented results show the close relationship of omentin-1 plasma levels to cholesterol synthesis and absorption. However, further research is needed to improve existing knowledge and to elucidate the pathophysiology of this hormone.

## Figures and Tables

**Table 1:** Characteristics of obese patients with diabetes mellitus type 1 during weight reduction programme

	*Phase I*	*Phase II*	*Phase III*	*Sig. I vs II*	*Sig. I vs III*
BMI (kg m^−2^)	33.2 (32.4; 33.6)	31.5 (30.4; 32.2)	32.7 (31.6; 32.9)	*P<0.001	*P<0.001
Waist circumference (cm)	108 (105; 110)	103 (94; 106)	105 (92; 110)	*P<0.001	*P<0.05
HbA1c (%, IFCC)	5.8 (5.5; 6.6)	5.7 (5.1; 6.2)	6.6 (6.1; 6.9)	NS	*P<0.05
Insulin per day (IU)	50.0 (39.3; 54.0)	37.5 (32; 40.3)	42.0 (39.3; 51.0)	*P<0.001	*P<0.001
Relative fat tissue mass (%)	36 (33.3; 37.8)	34.7 (32.7; 38.1)	34.3 (33.8; 34.9)	NS	NS
Relative lean tissue mass (%)	49.4 (43.1; 58.9)	53.4 (47.6; 54.8)	51.5 (49.6; 53.5)	NS	NS
Fat tissue index (kg m^−2^)	16.1 (14.8; 16.9)	14.4 (13.5; 15.7)	15.3 (14.1; 17.8)	*P<0.05	NS
Total cholesterol (mmol l^−1^)	4.95 (4.39; 5.57)	4.10 (3.88; 5.03)	4.91 (4.28; 5.47)	*P<0.05	NS
HDL cholesterol (mmol l^−1^)	1.30 (0.97; 1.59)	1.14 (1.03; 1.39)	1.49 (1.15; 2.01)	NS	*P<0.05
LDL cholesterol (mmol l^−1^)	3.35 (2.3; 3.7)	2.45 (2.02; 3.23)	2.71 (2.24; 3.11)	*P<0.05	NS
Triacylglycerols (mmol l^−1^)	1.28 (1.03; 1.59)	1.04 (0.91; 1.68)	0.83 (0.63; 1.41)	NS	NS
Free fatty acids (mmol l^−1^)	0.52 (0.35; 0.59)	0.40 (36; 0.68)	0.43 (0.29; 0.60)	NS	*P<0.05
Squalene (μmol l^−1^)	2.05 (1.23; 2.41)	2.09 (1.80; 2.51)	1.49 (0.60; 2.84)	NS	NS
Lathosterol (μmol l^−1^)	8.15 (6.46; 10.25)	9.37 (7.55; 9.80)	5.44 (2.44; 6.09)	NS	*P*<0.001
Campesterol (μmol l^−1^)	13.53 (7.96; 16.46)	8.46 (6.22; 11.76)	5.68 (4.15; 7.53)	*P<0.05	*P<0.05
Sitosterol (μmol l^−1^)	8.08 (6.87; 11.76)	5.66 (4.61; 9.51)	7.42 (4.24; 8.70)	*P<0.05	NS
Lathosterol/cholesterol (μmol l^−1^ mmol l^−1^)	1.45 (1.28; 2.25)	1.82 (1.41; 2.37)	0.99 (0.53; 1.36)	*P<0.05	*P<0.05
Campesterol/cholesterol (μmol l^−1^ mmol l^−1^)	2.48 (1.56; 3.49)	1.88 (1.75; 2.89)	1.10 (0.95; 1.37)	*P<0.05	*P*<0.001
β-Sitosterol/cholesterol (μmol l^−1^ mmol l^−1^)	1.77 (1.01; 2.12)	1.48 (0.98; 1.75)	1.46 (0.87; 1.97)	NS	NS
Omentin (ng ml^−1^)	5.31 (3.72; 6.49)	5.05 (3.91; 7.32)	9.74 (9.11; 10.98)	NS	*P*<0.001

Abbreviations: BMI, body mass index; HDL, high-density lipoprotein; HbA1c, glycated haemoglobin; IFCC, International Federation of Clinical Chemistry; LDL, low-density lipoprotein; NS, non-significant; phase I, before weight reduction programme; phase II, after a month of weight reduction programme; phase III, after 12 months of weight reduction programme; Sig. I vs II, III, significance among phases I, II or III. *P, significant (*P*-value).

**Table 2:** Basic comparison of obese patients with diabetes mellitus type 1 and type 2

	*DM1—Phase I*	*DM2*	*Sig. DM1 vs DM2*
Age (years)	43 (36; 46)	46 (42; 48)	*P<0.05
BMI (kg m^−2^)	33.2 (32.4; 33.6)	35.1 (32.3; 37.1)	NS
Waist circumference (cm)	108 (105; 110)	111 (107; 113)	*P<0.05
HbA1c (%, IFCC)	5.8 (5.5; 6.6)	6.4 (5.8; 7.4)	*P<0.05
Relative fat tissue mass (%)	36 (33.3; 37.8)	38.5 (34.7; 49.2)	*P<0.001
Relative lean tissue mass (%)	49.4 (43.1; 58.9)	49.2 (33.1; 54.3)	*P<0.05
Omentin (ng ml^−1^)	5.31 (3.72; 6.49)	2.15 (1.32; 6.02)	*P=0.032

Abbreviations: BMI, body mass index; DM, diabetes mellitus; HbA1c, glycated haemoglobin; IFCC, International Federation of Clinical Chemistry; NS, non-significant; Sig., significance.

*P, significant (*P*-value).

Values represent medians (25% and 75% percentile).

**Table 3:** Correlation of circulating cholesterol metabolism markers with omentin plasma levels in obese patients with diabetes mellitus type 1

*Omentin (ng ml^−1^)*	*Spearman test* r	P*-value*
Total cholesterol (mol l^−1^)	0.081	0.62
HDL cholesterol (mol l^−1^)	0.44	0.0055
LDL cholesterol (mol l^−1^)	−0.20	0.231
Triacylglycerols (mol l^−1^)	−0.41	0.011
Skvalen (μmol l^−1^)	−0.073	0.66
Lathosterol (μmol l^−1^)	−0.34	0.033
Campesterol (μmol l^−1^)	−0.32	0.05
B-sitosterol (μmol l^−1^)	−0.23	0.17
Lathosterol/cholesterol (μmol l^−1^ mmol l^−1^)	−0.32	0.05
Campesterol/cholesterol (μmol l^−1^ mmol l^−1^)	−0.37	0.02
β-Sitosterol/ cholesterol (μmol l^−1^ mmol l^−1^)	−0.18	0.28
Squalene/cholesterol (μmol l^−1^ mmol l^−1^)	−0.13	0.42
Free fatty acids (mol l^−1^)	−0.34	0.041

Abbreviations: HDL, high-density lipoprotein; LDL, low-density lipoprotein; *r*, correlation coefficient.

**Table 4:** Correlation of changes in plasma omentin-1 levels with the changes in metabolic parameters (phases I–III) in obese patients with diabetes mellitus type 1

*Omentin (ng ml^−1^)*	*Spearman test* r	P*-value*
HbA1c (% IFCC)	−0.72	0.008
Total cholesterol (mol l^−1^)	−0.19	0.52
HDL cholesterol (mol l^−1^)	0.24	0.44
LDL cholesterol (mol l^−1^)	−0.13	0.656
Triacylglycerols (mol l^−1^)	−0.64	0.022
Skvalen (μmol l^−1^)	0.44	0.14
Lathosterol (μmol l^−1^)	0.42	0.17
Campesterol (μmol l^−1^)	0.48	0.11
B-sitosterol (μmol l^−1^)	0.36	0.23
Lathosterol/cholesterol (μmol l^−1^ mmol l^−1^)	0.47	0.12
Campesterol/cholesterol (μmol l^−1^ mmol l^−1^)	0.42	0.17
β-Sitosterol/cholesterol (μmol l^−1^ mmol l^−1^)	0.41	0.17
Free fatty acids (mol l^−1^)	0.55	0.06

Abbreviations: HbA1c, glycated haemoglobin; HDL, high-density lipoprotein; IFCC, International Federation of Clinical Chemistry; LDL, low-density lipoprotein; *r*, correlation coefficient.
